# The Royal College of Psychiatrists Child and Adolescent Psychiatry Surveillance System for rare events and disorders: highlighting the need for an international network for surveillance

**DOI:** 10.1192/bji.2024.36

**Published:** 2025-02

**Authors:** Muthukrishnan Venkatesan, Eleanor Smith, Marinos Kyriakopoulos, Aditya Narain Sharma

**Affiliations:** 1 Speciality Trainee in Child and Adolescent Psychiatry, Newcastle and Gateshead Children and Young People's Service – Newcastle and Gateshead, Cumbria, Northumberland, Tyne and Wear NHS Foundation Trust, Newcastle upon Tyne, UK; 2 Consultant Child and Adolescent Psychiatrist, Complex Neurodevelopmental Disorders Service, Cumbria, Northumberland, Tyne and Wear NHS Foundation Trust, Newcastle upon Tyne, UK; 3 Assistant Professor in Child and Adolescent Psychiatry, 1st Department of Psychiatry, National and Kapodistrian University of Athens, Athens, Greece; 4 Consultant Child and Adolescent Psychiatrist, South London and Maudsley NHS Foundation Trust, London, UK; 5 Visiting Senior Lecturer, Department of Child and Adolescent Psychiatry, Institute of Psychiatry, Psychology and Neuroscience, King's College London, London, UK; 6 Clinical Senior Lecturer and Honorary Consultant in Child and Adolescent Psychiatry, Translational and Clinical Research Institute, Newcastle University, Newcastle on Tyne, UK. Email: aditya.sharma@ncl.ac.uk

**Keywords:** Child and adolescent psychiatry, epidemiology, longitudinal data, rare disorders and events, global mental health

## Abstract

Commonly occurring mental health disorders have been well studied in terms of epidemiology, presentation, risk factors and management. However, rare or uncommon mental health disorders and events are harder to study. One way to do this is active surveillance. This article summarises how the Royal College of Psychiatrists Child and Adolescent Psychiatry Surveillance System was developed, as well as the key studies that have used the system and their impact, to make the case for a wider international surveillance unit for child and adolescent psychiatry. Keeping this surveillance active in different populations across the globe will add to existing knowledge and understanding of these uncommon disorders and events. This will in turn help in developing better frameworks for the identification and management for these disorders and events. It will also facilitate the sharing of ideas regarding current methodology, ethics, the most appropriate means of evaluating units and their potential applications.

## Global burden of mental disorders in children and young people

Globally, the prevalence of mental health disorders in children and young people has contributed to the increasing burden of these disorders. One in five children in England has a probable mental health disorder, and this increases to one in four among 17–19-year-olds.^1^ Mental health disorders in children and young people cause significant distress and can affect multiple areas of functioning (e.g. education, family life, peer relationships) and physical health, all of which may have implications for individual developmental trajectories. Around half of children and young people diagnosed with mental health disorders will have ongoing difficulties in adulthood.^2^

## Rare mental health disorders

Commonly occurring mental health disorders have been well studied in terms of epidemiology, presentation, risk factors and management. However, rare or uncommon mental health disorders (defined as those that occur in fewer than one in 2000 individuals)^3^ cannot be well studied using community research. Some examples of these rare disorders include childhood eating disorders, paediatric onset bipolar disorder and childhood conversion disorder. Classification of disorders is constantly evolving, and any changes may lead to the inclusion of new, possibly rare, disorders in the classification systems which could be studied with the use of surveillance methodology. Although rare, these disorders can have a significant impact on children, young people and their families, as well as on mental health resources. The low numbers of these rare disorders in the population often cause delays in timely recognition and management.^4^ To study these disorders in detail and to generate scientific literature for clinicians, alternative methods of identification are needed.

## Surveillance epidemiology

Active surveillance is an established method of generating accurate and timely data on rare health conditions and events which are difficult to identify using conventional methods. Although it requires more resources than conventional approaches, this method is likely not to miss any cases and provides more comprehensive and valid data regarding the condition under study; this is particularly important when studying rare conditions.^5^ Rare paediatric disorders and events have been studied by the British Paediatric Surveillance Unit (BPSU) since the 1980s. The BPSU has provided useful data that has influenced vaccine and national screening policies, clinical research and practice on the ground. The BPSU was also instrumental in encouraging the development of similar surveillance systems across the globe.^6^ This paved the way for the establishment of the Child and Adolescent Psychiatry Surveillance System (CAPSS; https://www.rcpsych.ac.uk/improving-care/ccqi/research-and-evaluation/current-research/capss). This article summarises how CAPSS was developed, together with key studies that have used the system and their impact, to make the case for development of a wider international surveillance unit for child and adolescent psychiatry.

## Development of CAPSS

In 2005, a pilot system for CAPSS was initiated to study early-onset eating disorders. The response to this study was promising, with more than 95% of consultants in child and adolescent psychiatry (CCAPs) supporting the need for the system and agreeing to continue to contribute to the surveillance. This enthusiasm from clinicians about a system for surveillance of rare child and adolescent mental health disorders led to CAPSS being officially established in 2009.^7^ CAPSS, which is hosted by the Royal College of Psychiatrists, aims to:

- facilitate epidemiological surveillance and research into rare child and adolescent mental health disorders and events;
- increase awareness among the medical profession and the public about these disorders and events, as well as their impact on children and adolescents;
- enable psychiatrists to participate in surveillance of such conditions;
- inform clinical strategy and public health policy;
- respond in a timely manner to clinical and public health concerns.

## Planning and execution of the studies

CAPSS maintains a database of CCAPs working across the UK and Republic of Ireland. Researchers wishing to use the system submit proposals to the CAPSS executive committee for review. Once the proposal has been approved, researchers seek relevant ethics and governance clearances. An electronic reporting card with case definitions for all the disorders currently under study is emailed to all the CCAPs in the database each month. CAPSS informs the researchers of any notifications so that they can send out an initial questionnaire to establish caseness and, where relevant, follow-up questionnaires.^7^

## Impact of CAPSS studies

The data gathered from the study on early-onset eating disorders have highlighted the need for a developmental framework for diagnosis which is reflected in the DSM-5.^8^ This study has also encouraged more liaison between paediatricians and psychiatrists, which is critical to safe and effective clinical care. The incidence rates from the study on narrow-phenotype paediatric bipolar disorder provided information about the need for specialist services for this population.^9^ Studies on transitioning care for attention-deficit hyperactivity disorder to adult services, gender dysphoria and childhood disintegrative disorder have also been completed and have provided insights for planning of service provision.^7^ The most recent study on avoidant/restrictive food intake disorder (ARFID) was the largest to date. The incidence rates reported by the study indicated that ARFID may not be as rare as it was thought to be, and that it may require the development of specific clinical pathways.^10^ Details of the studies completed using CAPSS are provided in Table 1.

**Table 1:** CAPSS studies to date and key findings

Study name	Surveillance period	Study findings
Childhood Eating Disorders: British National Surveillance Study	Mar 2005 to May 2007	208 individuals were identified, with an estimated incidence of 3.01/100 000.
A surveillance study of childhood conversion disorder in the UK and Ireland	Oct 2008 to Oct 2009	204 cases were identified, giving a 12-month incidence of 1.30/100 000 (95% CI 1.11–1.52). The most common symptoms were motor weakness and abnormal movements.
A surveillance study of paediatric bipolar I disorder in the UK and Republic of Ireland	Sep 2009 to Sep 2010	151 possible cases were reported during the study period. Of these, 33 cases met the analytic case definition of NPBD, with an estimated annual incidence of 0.59/100 000 (95% CI 0.41–0.84).
A surveillance study of childhood-onset non-affective psychosis in the UK and Republic of Ireland	Sep 2010 to Sep 2011	15 cases with a provisional diagnosis of non-affective psychosis were reported. 1-year outcome data were obtained for 12 individuals, eight of whom met the criteria for schizophrenia or related diagnosis, equating to an estimated incidence of 0.21 per 100 000.
Investigating the burden of gender identity disorder in children and adolescents: a surveillance study of incidence, clinical presentation, comorbidities and natural history	Nov 2011 to Nov 2012	There were 230 confirmed cases during the reporting period, excluding duplicates (98 males), with a median age at diagnosis of 14.68 (interquartile range 12.1–15.31) years. Approximately two-thirds of cases aged less than 12 years were male (36 of 57 cases), with females comprising almost two-thirds of cases aged 12–15 years (111 of 173 cases). At least one comorbid mental health condition was present in 52% of 12–15-year-olds and 26% of under 12s.
The cost and cost-effectiveness of community-based models of care for child and adolescent anorexia nervosa	Feb 2015 to Feb 2016	The results of this study suggest that initial assessment in a specialist eating disorders service for young people with anorexia nervosa may have a higher probability of being cost-effective than initial assessment in generic CAMHS, although the associated uncertainty makes it hard to draw firm conclusions.
Childhood Disintegrative Disorder Surveillance Study	Nov 2016 to Nov 2017	19 cases (median age 4.7 years) were identified. All cases presented with loss of language and social skills, 50% experienced loss of bladder and bowel control, 88% experienced loss of adaptive skills, and 93% experienced loss of academic skills. The majority had significant internalising and externalising symptoms.
Where do they go? The transition of young people with ADHD across the age boundary of CAMHS	Nov 2015 to Nov 2016	For those aged 17–19 years, the incidence rate of transition need was 202–511 per 100 000 people per year, with successful transition of 38–96 per 100 000 people per year. Eligible young people with ADHD were mostly male (77%) with a comorbid condition (62%). Only 6% met optimal transition criteria.
A surveillance study of early onset depression in children as young as 3 years of age in the UK	Jan 2019 to Jan 2020	29 cases met the analytical case definition. Minimum incidence was 0.28/100 000 (95% CI 0.19–0.41). Age range 9.75–12.83 (11.47 ± 0.96) years, with 15 males and 14 females.
A surveillance study of Sydenham's chorea in children aged 0–16 years in the UK and Ireland	May 2019 to Nov 2020	Over 24 months, 72 reports were made via the BPSU, of which 43 met the surveillance case definition of suspected or confirmed Sydenham's chorea, which translates to an estimated paediatric service-related incidence rate of new Sydenham's chorea cases of 0.16 per 100 000 children aged 0–16 per year in the UK. No cases reported via CAPSS.
Incidence of ARFID in children and young people	Mar 2021 to Mar 2022	Identified 319 children and adolescents with ARFID. LCA revealed four distinct classes: fear subtype, lack of interest subtype, sensory subtype and combined subtype. The probability of being classified as these were 7.2% (*n* = 23), 25.1% (*n* = 80), 29.5% (*n* = 94) and 38.2% (*n* = 122), respectively.
‘Far Away from Home’ – mapping and understanding the impact of current practices for accessing in-patient care for adolescents with mental health difficulties: mixed methods study	Feb 2021 to Feb 2022	Data were collected about 290 admissions (follow-up rate 99% (288 of 290); sample were 73% female, mean age 15.8 years). The estimated adjusted yearly incidence of at-distance admission was 13.7–16.9 per 100 000 young people 13–17 years old. 38% were admitted >100 miles from home and 8% >200 miles. The most common diagnoses at referral were depression (34%) and autism spectrum disorder (20%).

ADHD, attention-deficit hyperactivity disorder; ARFID, avoidant/restrictive food intake disorder; BPSU, British Paediatric Surveillance Unit; CAMHS, child and adolescent mental health services; CAPSS, Child and Adolescent Psychiatry Surveillance System; LCA, latent class analysis; NPBD, narrow phenotype bipolar disorder.

## Challenges and way forward

Despite promising results from the studies, CAPSS is not without challenges. It has been noted from surveys that patients with certain child and adolescent psychiatry conditions present to community paediatricians rather than CCAPs;^11^ examples include neurodevelopmental disorders, psychiatric manifestations of neurological conditions and emotional difficulties, especially in those under 5 years old. Individuals with some of the emotional disorders also initially present to general practitioners.

Within child and adolescent mental health services (CAMHS), the nature of involvement of child and adolescent psychiatrists in different clinical scenarios varies considerably.^12^ Different CAMHS have different referral criteria, and the accepted referrals are distributed among different clinical pathways that may or may not include input from a CCAP. There are also child and adolescent psychiatrists within the teams who are trainees or specialist/associate grades and do not currently report to CAPSS. There is a need to keep in mind the possibility of cases being missed from surveillance and to consider strategies to prevent this. Finally, the development of such a process presupposes the existence of a mental health system which allows contact and coordination of all specialists within a region or country.

As well as the challenges described above, a major limitation of the current system is the geographical restriction in gathering of the data. This, combined with the innate rarity of the disorders being studied, raises the probability of insufficient data being collected. Moreover, without data from different countries and cultures, the cross-cultural generalisability of data is limited.

## The need for a global network for surveillance in child and adolescent psychiatric disorders

The BPSU has set an example globally and led to the development of 12 other paediatric surveillance systems around the world. These have now collaborated to form the International Network of Paediatric Surveillance Units, which covers over 50 million children and has studied more than 150 rare disorders. With growing awareness of child mental health disorders across the globe, CAPSS can pave the way for the establishment of a similar international system of surveillance.

Any such system and the studies using it would need to acknowledge that some mental health disorders have strong cultural influences, and so clinical presentations, diagnostic thresholds and perceptions about the disorders may vary. Keeping this surveillance active in different populations across the globe will add to existing knowledge and understanding of these uncommon disorders and events. This will in turn help in developing better frameworks for the identification and management for these disorders and events. It will also facilitate the sharing of ideas regarding current methodology, ethics, the most appropriate means of evaluating units and their potential applications. Overall, this surveillance system may improve the knowledge of child psychiatrists about rare disorders and lead to improved diagnostic validity for these disorders and the development of treatment guidelines.

## Data Availability

Data availability is not applicable to this article as no new data were created or analysed in this study.
